# Analysis of the cause of missed diagnosis in mpMRI/TRUS fusion-guided targeted prostate biopsy

**DOI:** 10.1186/s12894-022-01021-8

**Published:** 2022-05-06

**Authors:** Fan Zhang, Shun Zhang, Haifeng Huang, Qing Zhang, Shengjie Zhang, Shiwei Zhang, Hongqian Guo

**Affiliations:** Department of Urology, Nanjing Drum Tower Hospital, The Affiliated Hospital of Nanjing University Medical School, 321 Zhongshan Road, Nanjing, 210008 Jiangsu China

**Keywords:** Transrectal ultrasound, Magnetic resonance, Prostate cancer, Targeted prostate biopsy, Missed diagnosis

## Abstract

**Objectives:**

To investigate the causes of missed diagnosis in mpMRI/TRUS fusion-guided targeted prostate biopsy.

**Methods:**

The clinical data of 759 patients who underwent transperineal prostate biopsy from March 2021 to June 2021 at Nanjing DrumTower Hospital were retrospectively analyzed. Twenty-one patients had MRI contraindications. Ultimately, 738 patients completed mpMRI/TRUS fusion-guided targeted prostate biopsy + 12-core transperineal systematic biopsy after mpMRI and PI-RADS scoring. The pathological diagnoses from targeted and systematic biopsy were compared to evaluate and analyze the reasons for missed diagnoses in targeted biopsy.

**Results:**

A total of 388 prostate cancer patients were identified, including 37 (9%) missed diagnoses with targeted biopsy and 44 (11.34%) with systematic biopsy. Between the target biopsy missed diagnosis group and not missed diagnosis group, there was no significant difference in age (71.08 ± 7.11 vs. 71.80 ± 7.94), but PSA (13.63 ± 12.41 vs. 54.54 ± 177.25 ng/ml), prostate volume (61.82 ± 40.64 vs. 44.34 ± 25.07 cm^3^), PSAD (0.27 ± 0.28 vs. 1.07 ± 2.91), and ISUP grade [1(1) vs. 3(2)] were significantly different. The pathological results of the 37 targeted biopsy missed diagnoses were recompared with MRI: 21 prostate cancers were normal on MRI; 9 cancer areas were abnormal on MRI; and 7 cancer areas on MRI were PI-RADS 3.

**Conclusions:**

Early prostate cancer, large prostate, effect of local anesthesia, doctor–patient cooperation, MRI diagnosis, and operator technology were possible factors for missed diagnosis in targeted biopsy. Improvements imaging technology, greater experience, and personalized biopsy may lead to an accurate pathological diagnosis.

## Introduction

The incidence of prostate cancer is the highest among all male cancers in the United States [1] and is increasing in China every year [2]. Prostate biopsy is the main method for diagnosing prostate cancer. The guided types of prostate biopsy include finger guided, transrectal ultrasound (TRUS)-guided, and magnetic resonance image (MRI)-guidedbiopsies; the former two types are also used to guide systematic biopsy (SB) [3]. The 2020 European Association of Urology (EAU) guidelines on prostate cancer [4] and 2021 National Comprehensive Cancer Network (NCCN) Guidelines [5] has recommended multiparameter magnetic resonance imaging (mpMRI) as an important imaging method for detecting prostate cancer. mpMRI/TRUS fusion-guided targeted biopsy (TB) has become the main method because of the accuracy of MRI and flexibility of TRUS. In this study, we sought to identify the causes of and strategies for avoiding TB missed diagnosis to improve the positive rate of TB by analyzing 759 patients who underwent transperineal prostate biopsy.

## Objects and methods

### Objects

A total of 759 patients who received transperineal prostate biopsy from March 2021 to June 2021 in Nanjing DrumTower Hospital, aged from 42 to 95 (70.18 ± 8.04) years. Inclusion criteria, fulfil one of the following conditions: (1) abnormal digital rectal examination, (2) abnormal imaging examination (including abnormal MRI, ultrasound, PET, CT), (3) prostate specific antigen (PSA) > 10 ng/mL, (4) PSA 4–10 ng/mL and Prostate Imaging and Reporting and Data System (PI-RADS) score ≥ 3 according to MRI. Patients with contraindications, including coagulation dysfunction, infection, severe cardiopulmonary insufficiency, etc., were excluded.

### Method

#### mpMRI

Patients were scanned with a Philips Achieva 3.0T MRI scanner and the following scanning sequences: T1-weighted imaging (T1WI), T2-weighted imaging (T2WI), diffusion weighted imaging (DWI, b-value 1500), apparent diffusion coefficient (ADC), and dynamic contrast enhanced (DCE) imaging. All MR images were analyzed by two senior radiologists according to the prostate MRI section of the 2020 European urology guidelines [4] and graded by the PI-RADS version 2.1 [6] for suspected prostate cancer area: a score of 1 was considered benign, 2 likely benign, 3 between benign and malignant, 4 likely malignant, and 5 highly suspected malignant.

#### Biopsy

An Italian ESAOTE MyLabTwice ultrasonic diagnostic system was used to perform the biopsies. A real-time virtual sonography (RVS) image fusion system was adopted for mpMRI/TRUS image fusion, which can fuse MRI images with real-time TRUS images and display the target lesion from the MRI scan on the TRUS images in real time. A 18 G × 20 cm automatic biopsy needle by Gallini company in Italy was used to extract a specimen of length 20 mm. With the patient in the lithotomy position, after local perineal anesthesia with 1% lidocaine, one lesion with a PI-RADS score ≥ 3 on MRI was punctured with 2–4 needles, followed by standard 12-core transperineal SB. For patients with lesions with a PI-RADS score ≤ 2 or no lesion on MRI, 12-core SB was performed, which were classified as TB negative. Antibiotics were administered to prevent infection after the operation.

#### Histopathology

Any prostate cancer diagnosis was considered. Histopathology of prostate cancer was illustrated by International Society of Urological Pathology (ISUP). A 5-tier ISUP Grading System was established on the basis of Gleason grades, with grade 1 tumors being Gleason score (GS) ≤ 6; grade 2 being GS 3 + 4 = 7; grade 3 being GS 4 + 3 = 7; grade 4 being GS 4 + 4 = 8, 3 + 5 = 8 or 5 + 3 = 8; and grade 5 being GS 9–10. [7].

### Statistical methods

The statistical software SPSS 17.0 was used to analyze and process the data. Based on the pathological diagnosis, differences were analyzed between TB and SB. Counted data are indicated by % and were compared with the Χ^2^, categorical variable are indicated by median (interquartile range) and were compared with rank sum test, and continuous data are indicated by ${\overline{\text{X}}}$ ± S and were compared with the t test. *P* < 0.05 was considered statistically significant.

## Results

Among the 759 patients, prostate cancer was detected in 403 patients, with a positive rate of 53.10%. Twenty-one of these patients did not undergo MRI due to metal implants or claustrophobia and were not included in the following analysis.

A total of 738 patients completed TB + SB after mpMRI, and prostate cancer was detected in 388 patients, with a positive rate of 52.57% (Table 1). According to the biopsy pathology, patients were divided into four groups: 37 patients who were TB negative and SB positive (TB− & SB+ group), 44 TB positive and SB negative (TB+ & SB− group), 307 TB positive and SB positive (TB+ & SB+ group), and 350 TB negative and SB negative (TB− & SB− group). The TB− & SB+ patients were called the TB missed diagnosis group (TB-MD); the TB+ & SB− patients were called the SB missed diagnosis group (SB-MD); the TB+ & SB− and TB+ & SB+ patients were grouped into the TB not-missed diagnosis group (TB-NMD), with a total of 351; and the TB− & SB+ and TB+ & SB+ patients were grouped into the SB not-missed diagnosis group (SB-NMD), with a total of 344 (Table 2).

**Table 1:** Patient demographics, number of core, and biopsy findings (overall cohort)

	Total(n = 738)	Histopathology	
		PCa	Negative
Age	70.04 ± 7.96	71.73 ± 7.88	68.17 ± 7.63
PSA (ng/mL)	32.26 ± 128.02	50.66 ± 163.73	11.86 ± 14.00
PV (cm^3^)	58.06 ± 35.26	51.73 ± 27.90	65.07 ± 41.63
Cores			
TB	2.64 ± 1.08	2.86 ± 1.17	2.43 ± 0.95
SB	12.00 ± 0.00	12.00 ± 0.00	12.00 ± 0.00
ISUP			
1		n = 76	
2		n = 87	
3		n = 87	
4		n = 103	
5		n = 35	

Prostate volume (PV) = π/6 × width × height × length

**Table 2:** Missed diagnosis in TB and SB

	TB	SB	*P*
Missed diagnosis	37	44	–
Not missed diagnosis	351	344	–
Total	388	388	–
Missed diagnosis rate	9.54%	11.34%	0.41

A comparison between the TB-MD group and the TB-NMD group is shown in Table 3.

**Table 3:** Differences between TB-MD group and TB-NMD group

	Age	PSA (ng/mL)	PV (cm^3^)	PSAD	ISUP
TB-MD	71.08 ± 7.11	13.63 ± 12.41	61.82 ± 40.64	0.27 ± 0.28	1 (1)
TB-NMD	71.80 ± 7.94	54.54 ± 177.25	44.34 ± 25.07	1.07 ± 2.91	3 (2)
*P*	0.60	0.00	0.02	0.00	0.00

Prostate specific antigen density (PSAD) = PSA/PV

The pathological results of the TB-MD group were compared with MR image again: 21 patients’ any area of MR images were normal; 9 patients’ cancer areas detected by SB were TB areas on MR image; and 7 patients’ cancer areas detected by SB were corrected to PI-RADS 3 on MR image.

In the TB+ & SB+ group, ISUP grade of the TB sample was greater than or equal to that of the SB for 276 patients, accounting for 89.90% of the patients in this group. Analyzing the SB-positive locations on the MR images, 45 patients had SB-positive areas with non-TB-positive areas, which means that TB missed prostate cancer lesions in these cases, accounting for 14.66%. 24 patients’ cancer lesions missed by TB were normal on MRI; 10 patients’ cancer lesions missed by TB were other TB-negative areas; and 11 patients’ cancer areas detected by SB were corrected to PI-RADS 3 on MRI. The ISUP grade of the cancer lesions with TB missed diagnoses was 1 (1), and the ISUP grade of cancer lesions with TB diagnoses was 3 (2), *P* = 0.00. The ISUP grade of the TB lesions was greater than or equal to that of the TB missed diagnosis lesions, accounting for 93.33%.

## Discussion

Prostate biopsy, as the gold standard for prostate cancer diagnosis, can be performed in a guided manner, ranging from finger guidance to TRUS guidance. With the development of imaging technology, mpMRI has shown obvious advantages in prostate cancer diagnosis, especially with implementation of the PI-RIDS, allowing a quantified and standardized diagnosis. Quentin et al. [8] reported that MRI can be used to directly guide prostate biopsy; however, as MRI cannot be performed on patients with metal implants, involves a long scanning time and cannot provide real-time guidance, and requires an expensive, complex, and long biopsy procedure, this method has not been used widely. The emergence of image fusion technology perfectly combines the convenience, ease of operation and low cost of TRUS with the high sensitivity of mpMRI for prostate cancer, addressing the lack of random sampling in systematic puncture biopsy, and has been gradually implemented in clinical practice.

Leveraging TB technology and the MRI screening of patients with PSA 4–10 ng/mL, the positive rate of all 759 patients was 53.10%, higher than that previously reported for SB (positive rate 38.2%) [9]. Some patients were contraindicated for MRI and thus could not undergo mpMRI-TRUS fusion TB. In this study, local anesthesia, transperineal free-arm biopsy was performed, which can be carried out on a large scale for outpatient or daytime patients. The operation time is approximately 15 min and has the advantage of imparting a low risk of bleeding and infection. Patients generally tolerate the procedure well, and the prostate can be punctured with no blind spot.

In this study, the missed diagnosis rate of TB was slightly lower than that of SB, but there was no significant difference; both TB and SB missed approximately 10% of prostate cancers, so TB + SB is currently the best way to perform prostate biopsy.

In the TB missed diagnosis group, a TB missed diagnosis was not significantly associated with patient age but was significantly associated with lower PSA, larger prostate volume, lower PSAD and lower ISUP grade, so early prostate cancer and large prostate volume were more likely to cause TB missed diagnosis.

Among the 37 patients with TB missed diagnoses, the MRIs of 21 prostate cancers diagnosed by SB only showed no abnormalities, which is associated with the sensitivity of MRI; previous studies have concluded that MRI had higher sensitivity to high-risk PCa and tumors longer than 5 mm in diameter [10]. The PCa areas sampled by SB in 9 patients were TB target areas, suggesting that TB did not hit the MRI target area. The possible reasons for this are as follows: (1) Fusion error: different bladder filling state during MRI examination and biopsy, deformation caused by insertion of rectal prostate probe, and operator’s judgment of the datum plane can all affect the fusion of the MR and TRUS images. Consequently, the target lesion region displayed on the TRUS image will be offset from the actual target lesion region on MRI, causing the actual target lesion to be missed. (2) Involuntary patient movement due to pain and discomfort during biopsy [11], causing displayed target lesion deviation. (3) The target lesion area shown on MRI contained both inflammation and cancer tissue, and only the inflammation area was punctured, which resulted in a missed cancer diagnosis; (4) The target lesion was too small to puncture. To reduce the probability of a missed diagnosis for such patients, the following methods could be considered: (1) The patient should empty the bladder before MRI examination and biopsy. (2) The median sagittal plane (urethral plane) should be used as the datum plane for fusion. (3) The local anesthesia should be improved by fully injecting the anesthetic into the area from the perineal skin to the apex region of the prostate, especially on both sides of the nerve vascular bundle. (4) Greater communication should be conducted with the patient before and during biopsy to ease the patient’s anxiety. (5) Larger lesions should be sampled with a greater number of TB needles. (6) The operator should have greater experience in puncturing, in particular, large prostates, small lesions, or lesions in difficult locations (pubic occlusion area, anterior urethral area, base and apex), Pepe et al. [12] found that mpMRI increased the diagnosis of PCa located in the anterior zone of the prostate, where were contained in difficult locations. In the other 7 patients, the PI-RADS scores were underestimated before biopsy, resulting in missed TB of actual PI-RADS 3 areas, which required a careful analysis of the MR images by MRI diagnostic doctors. Khosravi et al. [13] applied artificial intelligence (AI) to the evaluation of MR images and achieved good results. Through full communication between the radiologist and operator and puncturing all suspicious lesions, the positive rate of TB can be further improved.

The ISUP grade of TB and SB samples in the TB+ & SB+ group showed that TB achieved the highest grade in approximately 90% of patients. Prostate cancer is a multifocal tumor, which means there may be several cancer lesions in the prostate. Although every patient was diagnosed with prostate cancer in this group, 45 patients were SB-positive in the non-TB target areas, which means that they experienced missed diagnoses of their prostate cancer lesions. Comparing the missed lesions with the unmissed ones, it was found that the ISUP grade of the TB unmissed lesions was significantly higher than that of the TB missed lesions. Baco et al. [14] suggested that the index lesion be defined as the tumor lesion with the highest Gleason score, or if two or more lesions had the same Gleason score, the index lesion should be defined as the largest lesion. Liu [15] found that index lesions promoted the progression of prostate cancer; therefore, TB likely missed secondary lesions and instead sampled lesions that dominated the overall disease process. Comparing TB missed lesions with MRI in this subgroup and analyzing the causes of each lesion, we found similar results to the TB missed group.

TB has the advantage of requiring fewer needles, and most of the missed lesions were early prostate cancer or secondary lesions. This is similar to the findings of Zhang et al. [16], who found that TB could increase the detection rate of clinically significant prostate cancer while reducing the number of needles. Missed clinically nonsignificant prostate cancer does not necessarily have serious consequences for patients [17]. However, the disease will continue to progress, and these missed cases may delay the treatment of some patients. Pepe et al. [18] reported that 16.2% clinically significant prostate cancers missed by targeted fusion prostate biopsy and a PI-RADS score of 3 or greater. The aim of many existing prostate biopsy studies [19, 20] was to obtain the closest results to radical pathology, to avoid as many missed diagnoses of prostate cancer as possible, and to obtain the true ISUP grade and range of lesions. Patients with clinically significant prostate cancer require positive treatment; patients with clinically nonsignificant prostate cancer can choose the most appropriate intervention or wait for observation according to his or her actual situation and closely monitor the progress of prostate cancer. If a diagnosis is missed during biopsy for these patients, the best treatment opportunity in the progression of the disease may be lost.

The limitations of the present study include: this is a single-center retrospective study; primary objective is diagnoses of any prostate cancer rather than clinically significant prostate cancer; RVS image fusion system is non organ tracking MRI-TRUS fusion system. The patient and/or prostate movement during the biopsy, may significantly influence the precision of targeting. Further, the registration in 3D prostate volume of each biopsy track is difficult to evaluate, consequently it is difficult to confirm the real location of the biopsy cores. If improved fusion system can track organ (prostate) and record the real location of the biopsy cores, the MRI and biopsy findings could be correlated with pathologic large slice after radical prostatectomy, further study will help reduce missed diagnosis of prostate cancer more.

In summary, early prostate cancer, a large prostate, the effect of local anesthesia, patient cooperation, MRI reading, and skill of the operator are possible causes of TB missed diagnoses. Improved imaging technology, additional biopsy experience, improved fusion system, and personalized biopsy plans will help exploit the advantages of TB and further improve the positive rate to that of real prostate pathology.

## Data Availability

The datasets generated and analysed during the current study are not publicly available due to do not have consent from all patients to publish this data, but are available from the corresponding author on reasonable request.
